# Red blood cell distribution width-standard deviation but not red blood cell distribution width-coefficient of variation as a potential index for the diagnosis of iron-deficiency anemia in mid-pregnancy women

**DOI:** 10.1515/biol-2021-0120

**Published:** 2021-11-06

**Authors:** Yang Kai, Pan Ying, Yan Bo, Yu Furong, Chen Jin, Fu Juanjuan, Tian Pingping, Zhang Fasu

**Affiliations:** Department of Medical Technology, Anhui Medical College, Hefei, Anhui Province, 230601, People’s Republic of China; Department of Obstetrics and Gynecology, The Second Hospital of Anhui Medical University, Hefei, Anhui Province, 230601, People’s Republic of China

**Keywords:** RDW standard deviation, RDW coefficient of variation, iron deficiency anemia, mid-pregnancy women

## Abstract

The aim of this study was to compare the diagnostic values of red blood cell distribution width-coefficient of variation (RDW-CV) and red blood cell distribution width-standard deviation (RDW-SD) in mid-pregnancy women with iron deficiency anemia (IDA). To obtain the results, 115 mid-pregnancy women with IDA, defined as the IDA group, and 142 healthy mid-pregnancy women, selected as the control group, were enrolled in this study. Hematological parameters and ferritin concentrations in the serum were analyzed. The efficiency of RDW-CV and RDW-SD to distinguish IDA from mid-pregnancy women was evaluated using receiver operating characteristic (ROC) curves. The RDW-SD value in the IDA group was significantly higher than that in the control group (*p* < 0.05), while the RDW-CV value did not differ between them (*p* = 0.84). Significantly negative correlations were found between RDW-CV (*r* = −0.297, *p* = 0.001), RDW-SD (*r* = −0.404, *p* = 0.000), and serum ferritin in the IDA group but not in the control group. For the diagnosis of IDA, RDW-CV and RDW-SD produced areas under the ROC curves of 0.58 and 0.84. To conclude, our results suggest that RDW-SD, but not RDW-CV, can be used as a diagnostic index of IDA for mid-pregnancy women.

## 1 Introduction

Iron deficiency (ID) is known as one of the main nutritional deficiencies worldwide, and its occurrence can be observed in multiple medical conditions [1,2,3]. Iron deficiency anemia (IDA) during pregnancy can increase the risk of complications for the mother and fetus, including hypertension, puerperal infection, postpartum depression, premature labor, intrauterine growth retardation, perinatal mortality, and maternal mortality from hemorrhage [4]. Fortunately, ID and IDA during pregnancy are treatable and possibly preventable with iron supplementation. Therefore, screening for IDA is important in avoiding complications in pregnancy.

The red blood cell distribution width (RDW), including RDW-coefficient of variation (CV) and RDW-standard deviation (SD), as simple and widely available hematological indices, is easily acquired from a routine blood test and reflects the degree of heterogeneity of erythrocyte size [5]. Traditionally, RDW-CV is used to explore the cause of underlying anemia, especially for differential diagnosis of IDA [6,7]. However, Abdelrahman et al. found the poor diagnostic performance of RDW-CV in pregnant Sudanese women with IDA [8]. It is possible that RDW-CV (=1SD/MCV) is composed of mean corpuscular volume (MCV), which cannot mirror the small variations in red blood cell (RBC) size that occur in early iron deficiency [9]. Furthermore, unlike RDW-CV, RDW-SD eliminates the influence of MCV from RDW, which is calculated by the width of the erythrocyte volume distribution curve at a level 20% above baseline [10]. Although RDW-SD is considered another index reflecting RBC heterogeneity, up to now, data on the role of RDW-SD in screening for IDA during pregnancy are lacking. With this in mind, this study aimed to evaluate the diagnostic values of RDW-CV and RDW-SD in identifying IDA among mid-pregnancy Chinese women.

## 2 Materials and methods

### 2.1 Study population

The electronic medical records of 115 mid-pregnancy women with IDA in the Department of Obstetrics and Gynecology, the Second Hospital of Anhui Medical University, from January 2014 to December 2020 were retrospectively reviewed. IDA was defined according to the World Health Organization criteria: hemoglobin <10.5 g/dL and serum ferritin <15 µg/L. The exclusion criteria of this study were as follows: (1) cardiovascular disease; (2) hypertension; (3) acute or chronic infection; (4) immune disease; (5) kidney or liver dysfunction; (6) folate or vitamin B12 deficiency; and (7) WBC > 17.1 × 10^9^/L [8].


**Informed consent:** Informed consent has been obtained from all individuals included in this study.
**Ethical approval:** The research related to human use has been complied with all the relevant national regulations, institutional policies and in accordance with the tenets of the Helsinki Declaration, and has been approved by the Ethics Board of Anhui Medical College and the Second Hospital of Anhui Medical University.

### 2.2 Laboratory measurements

Serum ferritin concentrations were determined by electrochemiluminescence immunoassay (Roche Diagnostics, Mannheim, Germany). Routine blood tests, including RDW-CV and RDW-SD, were measured using an automated hematology analyzer, Sysmex XT-2000i (Sysmex, Kobe, Japan).

### 2.3 Statistical analysis

Statistical analyses were performed using SPSS software (version 19.0) and MedCalc software (version 11.4.2.0). The Shapiro–Wilk test was used to verify the normality of the data in this study. Data are shown as mean ± standard deviation (SD). Continuous variables were compared with the independent sample *t*-test or the Mann–Whitney *U* test. Correlation analysis between variables was computed using Spearman’s rank correlation coefficient analysis. Further, a multiple linear regression was used to analyze the relationship between serum ferritin and RDW-CV and RDW-SD. The diagnostic performance of RDW-CV and RDW-SD was estimated by ROC curve analysis. The *Z* statistic was used for pairwise comparison of the diagnostic performance between RDW-CV and RDW-SD in the diagnosis of IDA among mid-pregnancy women; *p* < 0.05 was considered statistically significant.

## 3 Results

### 3.1 Clinical characteristics of the subjects

A total of 142 healthy mid-pregnancy women (control group) and 115 mid-pregnancy women with IDA (IDA group) were enrolled in this study. Clinical characteristics of mid-pregnancy women are shown in Table 1. No statistically significant differences were found between the control group and IDA group regarding age and gestational age (both *p* > 0.05). Compared to the control group, the mean levels of ferritin, MCV, MCH, Hb, MCHC, RBC, and HCT were significantly lower in the IDA group (all *p* < 0.05).

**Table 1:** Clinical characteristics of subjects in this study

Variables	Control group	IDA group	*p*-value
Age (years)	30.39 ± 3.47	30.03 ± 3.81	0.43
Gestational age (weeks)	22.17 ± 2.40	22.30 ± 2.73	0.67
Ferritin (µg/L)	47.75 ± 20.11	11.11 ± 3.21	<0.05
MCV (fL)	94.75 ± 3.49	85.90 ± 5.84	<0.05
MCH (pg)	31.65 ± 1.25	27.27 ± 2.33	<0.05
Hb (g/dL)	11.87 ± 0.54	9.81 ± 0.47	<0.05
MCHC (g/L)	334.07 ± 6.81	325.26 ± 10.73	<0.05
RBC (10^12^/L)	3.76 ± 0.22	3.53 ± 0.32	<0.05
HCT (L/L)	0.36 ± 0.02	0.32 ± 0.02	<0.05
			

### 3.2 Comparison of RDW-CV and RDW-SD

**Figure 1:**
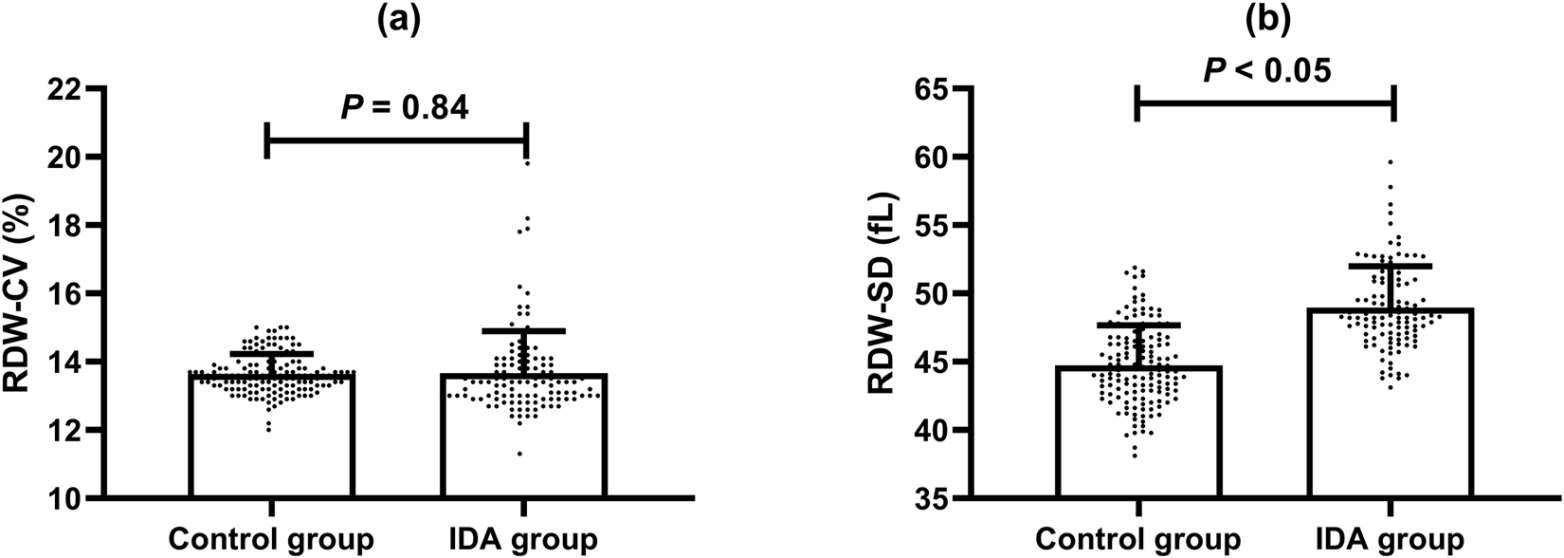
RDW-CV and RDW-SD in the control group (a) and the IDA group (b).

In the control group, all participants had normal values of RDW-CV (range: 12.0–15.0%) and RDW-SD (range: 38.1–51.9 fL). In the IDA group, 11 (10.75%) had RDW-CV values over 15% (the upper limit of the reference range), and 6 (5.23%) had RDW-SD values over 54 fL (the upper limit of the reference range). There were no significant differences in the RDW-CV values between the groups (13.64 ± 0.59 vs 13.66 ± 1.23, *p* = 0.84) (Figure 1a). However, RDW-SD values were significantly higher in the IDA group compared with the control group (48.96 ± 3.02 vs 44.75 ± 2.91, *p* < 0.05) (Figure 1b).

### 3.3 Correlation of RDW-CV, RDW-SD, and serum ferritin

**Figure 2:**
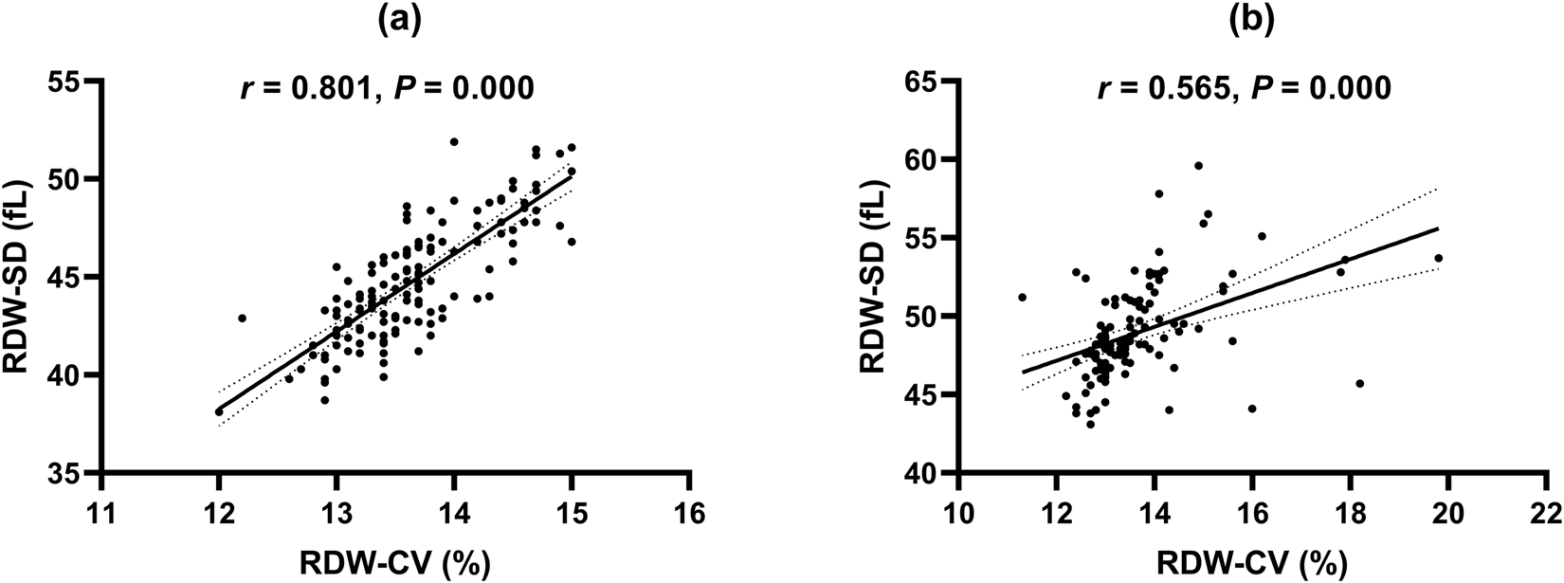
Correlations between RDW-CV and RDW-SD in the control group (a) and the IDA group (b).

**Figure 3:**
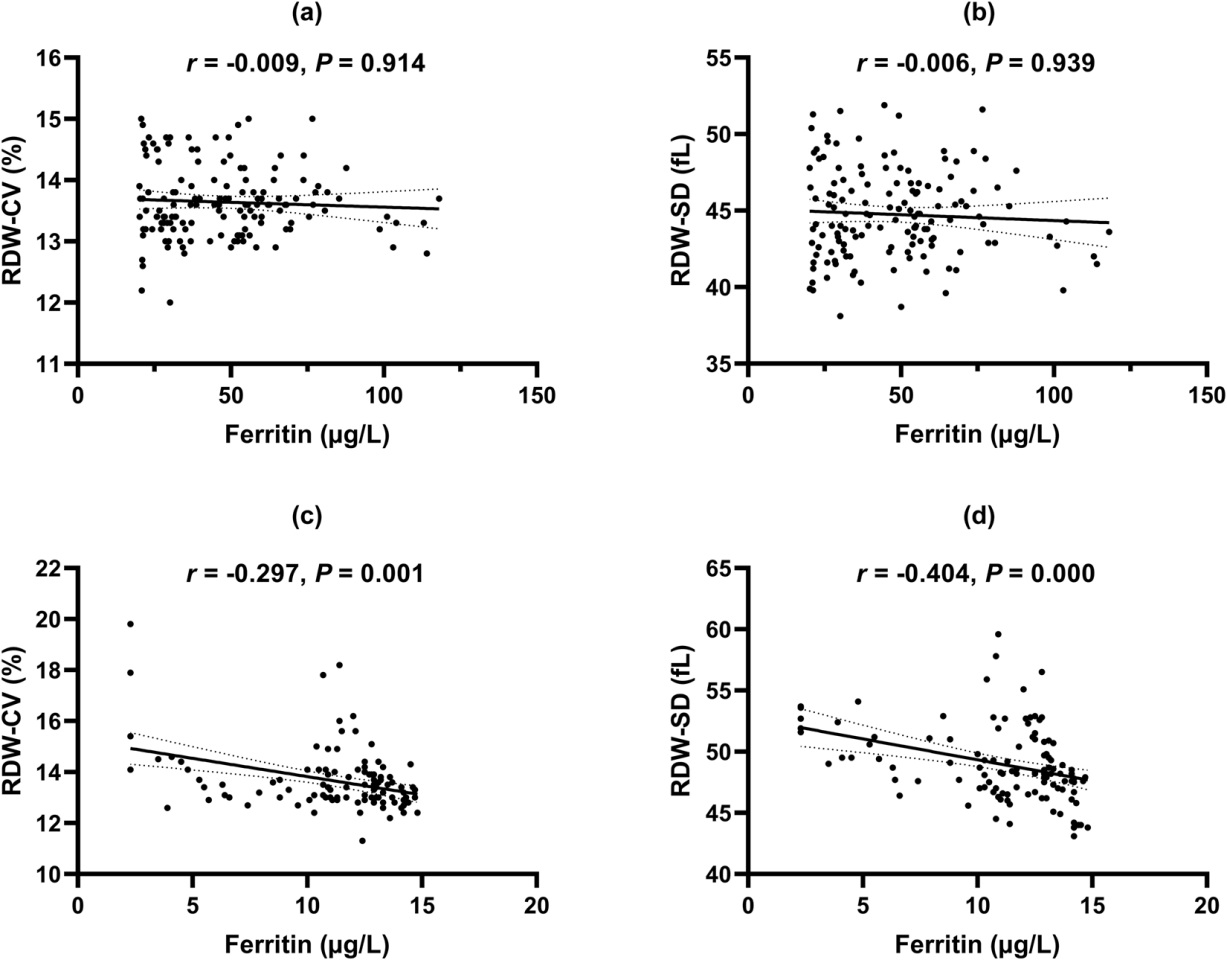
Correlations between RDW-CV, RDW-SD, and serum ferritin in the control group (a and b) and the IDA group (c and d).

A strong positive correlation between RDW-CV and RDW-SD was found in the control group (*r* = 0.801, *p* = 0.000, Figure 2a), whereas a less significant positive relationship between RDW-CV and RDW-SD was observed in the IDA group (*r* = 0.565, *p* = 0.000, Figure 2b). Serum ferritin concentrations are an important marker of IDA. We assessed the correlations between RDW-CV, RDW-SD, and serum ferritin. In the control group, neither RDW-CV (*r* = −0.009, *p* = 0.914, Figure 3a) nor RDW-SD (*r* = −0.006, *p* = 0.939, Figure 3b) had any significant correlation with serum ferritin. However, both RDW-CV (*r* = −0.297, *p* = 0.001, Figure 3c) and RDW-SD (*r* = −0.404, *p* = 0.000, Figure 3d) were negatively correlated with serum ferritin in the IDA group. Moreover, we performed linear regression analysis. The results showed that both RDW-CV (*β* = −0.214, *p* = 0.034) and RDW-SD (*β* = −0.327, *p* = 0.015) were independently associated with serum ferritin levels.

### 3.4 Diagnostic value of RDW-CV and RDW-SD for IDA

**Figure 4:**
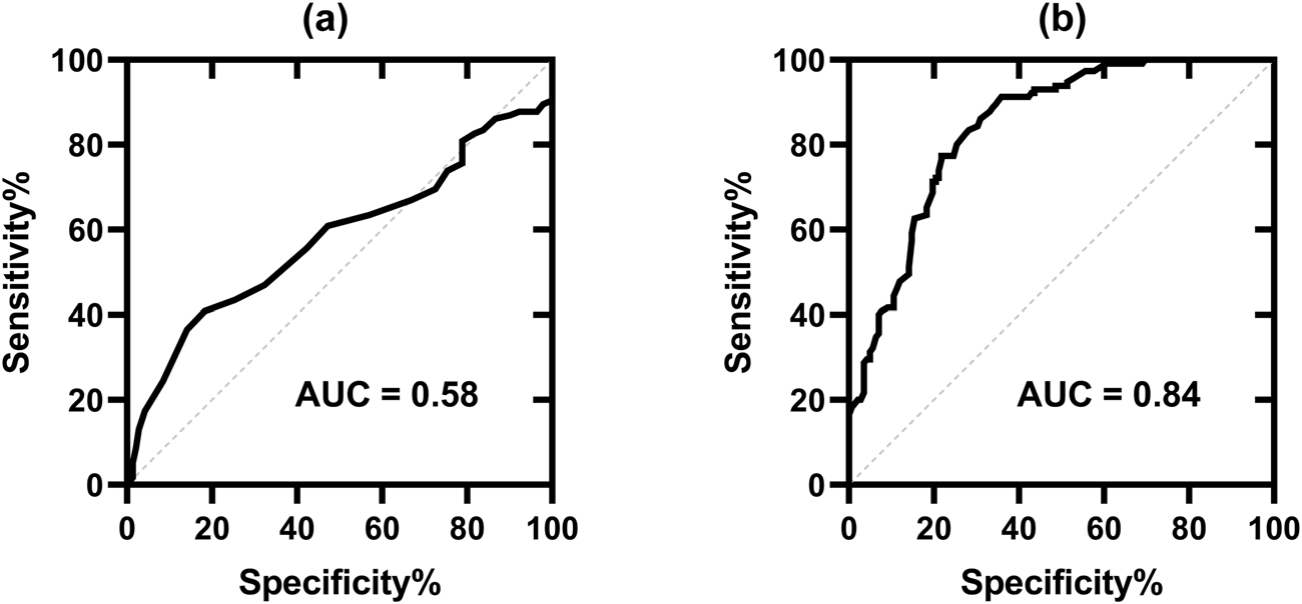
ROC curve for RDW-CV (a) and RDW-SD (b).

ROC curve analysis was performed using RDW-CV and RDW-SD to diagnose IDA in mid-pregnancy women. As shown in Figure 4a, the area under ROC curves (AUC) of RDW-CV was 0.58 (95% CI: 0.52–0.64), suggesting no role for RDW-CV in diagnosing IDA in mid-pregnancy women. As shown in Figure 4b, the AUC was 0.84 (95% CI: 0.79–0.89) for RDW-SD, which indicated that RDW-SD was superior to RDW-CV as a diagnostic index for IDA in mid-pregnancy women (*p* < 0.05). The sensitivity, specificity, positive predictive value, and negative predictive value of RDW-SD were 77.39, 78.17, 74.13, and 81.05%, respectively, at a cut-off point of 46.8 fL.

## 4 Discussion

In this study, we analyzed the values of RDW-CV and RDW-SD in Chinese mid-pregnancy women to investigate the possible benefits of RDW-CV and RDW-SD as diagnostic indices for IDA. The results of this study indicate that RDW-SD can be used in mid-pregnancy women to diagnose IDA, while RDW-CV has poor accuracy in the diagnosis of IDA.

Bone marrow aspiration is the gold standard for ID, but it is invasive, painful, costly, and time consuming [11]. Therefore, the identification of additional diagnostic indices for ID(A), especially if they can be readily, routinely, and cheaply obtained, is necessary. RDW is a routine complete blood count parameter that evaluates the variability in the size of erythrocytes in circulation. It is usually expressed as RDW-CV and RDW-SD, which is less influenced by other factors than the former [12,13]. In the last few years, numerous studies have consistently confirmed RDW-CV as a tool for the differential diagnosis of anemia. In this context, the relationship between RDW and IDA may be considered during pregnancy. A previous study performed in Bangladesh demonstrated that RDW-CV was useful for IDA diagnosis in pregnant women within the first 20 weeks of gestation [14]. By contrast, another study found that RDW-CV has a poor performance in diagnosing IDA among Sudanese pregnant women with a mean gestational age of 21.4 weeks [8]. Nevertheless, Tiwari et al. reported that RDW-CV correlated with serum ferritin concentrations and could help in the diagnosis of IDA among Indian women during the second and third trimesters of pregnancy [15]. These contrasting results suggest that the role of RDW-CV in the diagnosis of IDA during pregnancy requires further evaluation.

In this study, we observed that there was no significant difference in RDW-CV values between healthy mid-pregnancy women and mid-pregnancy women with IDA (*p* = 0.841). This result is partially in contrast to previously published results demonstrating elevated levels of RDW-CV in pregnant women with IDA [14]. This might be related to differences in the characteristics of the study population; on the other hand, different analytical methods might also account for divergent findings. Surprisingly, we found that the RDW-SD value of mid-pregnancy women with IDA was significantly higher than that of healthy mid-pregnancy women, suggesting that mid-pregnancy women with mild IDA had more significant RBC size variations than healthy mid-pregnancy women. Besides, a stronger correlation was observed between RDW-CV and RDW-SD in mid-pregnancy women with IDA compared to the two factors in healthy mid-pregnancy women (*r* = 0.565 vs *r* = 0.801). The earlier findings suggest the inconsistency of RDW-CV and RDW-SD in mid-pregnancy women with IDA.

Serum ferritin is the most specific marker reflecting total body iron stores and has been widely used in diagnosing IDA in pregnancy [16]. Our study demonstrated that both RDW-CV and RDW-SD were negatively correlated with serum ferritin in the IDA group but not in the control group. It should be noted that RDW-SD has a stronger negative correlation with serum ferritin compared to RDW-CV (*r* = −0.404 vs *r* = −0.297) in the IDA group. In addition, the result of multiple linear regression analysis showed that both RDW-CV and RDW-SD were independently correlated with serum ferritin levels. Importantly, standardized coefficients indicated that changes in serum ferritin levels have a stronger influence on RDW-SD (*β* = −0.327) than RDW-CV (*β* = −0.214), suggesting a potential role for RDW-SD in diagnosing IDA among mid-pregnancy women. A recent study proved that RDW-SD performs better than RDW-CV in the differential diagnosis of microcytic anemia. In that study, 16 of 23 discriminant formulas incorporating RDW-CV significantly improved thalassemia diagnosis accuracy by using RDW-SD instead of RDW-CV [7]. In our study, RDW-CV seems not to have a role in diagnosing IDA in mid-pregnancy women, which is consistent with Abdelrahman’s study results [8]. More importantly, we reported that RDW-SD could be used for the diagnosis of IDA in mid-pregnancy, with an AUC of 0.84, a sensitivity of 77.39%, and a specificity of 78.17%. Since RDW-SD more accurately reflects actual blood cell size variations, it is not surprising that RDW-SD performs better than RDW-CV in diagnosing IDA among Chinese mid-pregnancy women.

A few limitations should be taken into consideration. First, this was a single-center study with a small sample size. Second, we did not investigate the presence of thalassemia in mid-pregnancy women, which is associated with IDA. However, it is unlikely that thalassemia influenced the outcome of this study because of the low prevalence of thalassemia in our region. Third, predictive values of RDW-SD and RDW-CV in early pregnancy and late pregnancy were not investigated in this study due to the absence of available data; this requires further investigation.

To our knowledge, this is the first study to investigate the diagnostic capacity of RDW-SD for IDA in pregnant women. Our data suggest that RDW-SD may be an index of interest for correctly diagnosing IDA among mid-pregnancy women. RDW-SD is a noninvasive parameter that can be measured by automated blood cell analyzers without additional cost. Therefore, RDW-SD could serve as a screening indicator for IDA among mid-pregnancy women.

## References

[1] Gafter-Gvili A, Schechter A, Rozen-Zvi B. Iron deficiency anemia in chronic kidney disease. Acta Haematol. 2019;142:44–50. 10.1159/000496492 30970355

[2] Stein J, Connor S, Virgin G, Ong DE, Pereyra L. Anemia and iron deficiency in gastrointestinal and liver conditions. World J Gastroenterol. 2016;22:7908–25. 10.3748/wjg.v22.i35.7908 PMC5028806 27672287

[3] Mistry R, Hosoya H, Kohut A, Ford P. Iron deficiency in heart failure, an underdiagnosed and undertreated condition during hospitalization. Ann Hematol. 2019;98:2293–7. 10.1007/s00277-019-03777-w 31402406

[4] Esen UI. Iron deficiency anaemia in pregnancy: the role of parenteral iron. J Obstet Gynaecol. 2017;1:15–8. 10.1080/01443615.2016.1180505 27184678

[5] Łochowski M, Łochowska B, Chałubińska-Fendler J, Zawadzka I, Rębowski M, Kozak J. Prognostic value of red blood cell distribution width-standard deviation (RDW-SD) in patients operated on due to non-small cell lung cancer. J Thorac Dis. 2020;12:773–81. 10.21037/jtd.2019.12.94 PMC7138985 32274144

[6] Hu ZD, Lippi G, Montagnana M. Diagnostic and prognostic value of red blood cell distribution width in sepsis: a narrative review. Clin Biochem. 2020;77:1–6. 10.1016/j.clinbiochem.2020.01.001 31935355

[7] Hoffmann JJML, Urrechaga E. Role of RDW in mathematical formulas aiding the differential diagnosis of microcytic anemia. Scand J Clin Lab Invest. 2020;12:1–6. 10.1080/00365513.2020.1774800 32530320

[8] Abdelrahman EG, Gasim GI, Musa IR, Elbashir LM, Adam I. Red blood cell distribution width and iron deficiency anemia among pregnant Sudanese women. Diagn Pathol. 2012;7:168. 10.1186/1746-1596-7-168 PMC3538607 23206545

[9] Lian Y, Shi J, Nie N, Huang Z, Shao Y, Zhang J, et al. Reticulocyte hemoglobin equivalent (Ret-He) combined with red blood cell distribution width has a differentially diagnostic value for thalassemias. Hemoglobin. 2019;43:229–35. 10.1080/03630269.2019.1655440 31476929

[10] Liu L, Cao J, Zhong Z, Guo Z, Jiang Y, Bai Y, et al. Non-invasive indicators predict advanced liver fibrosis in autoimmune hepatitis patients. J Clin Lab Anal. 2019;33:e22922. 10.1002/jcla.22922 PMC6757115 31115929

[11] Dewan P, Dixit A, Gomber S, Kotru M, Banerjee BD, Tyagi V, et al. Serum and urinary hepcidin for diagnosing iron-deficiency anemia in under-5 children. J Pediatr Hematol Oncol. 2019;41:e216–20. 10.1097/MPH.0000000000001320 30334902

[12] Zhang FX, Li ZL, Zhang ZD, Ma XC. Prognostic value of red blood cell distribution width for severe acute pancreatitis. World J Gastroenterol. 2019;25:4739–48. 10.3748/wjg.v25.i32.4739 PMC6718036 31528098

[13] Li X, Chen Q, Bi X, Zhao J, Li Z, Zhou J, et al. Preoperatively elevated RDW-SD and RDW-CV predict favorable survival in intrahepatic cholangiocarcinoma patients after curative resection. BMC Surg. 2021;21:105. 10.1186/s12893-021-01094-6 PMC7919078 33648470

[14] Sultana GS, Haque SA, Sultana T, Rahman Q, Ahmed AN. Role of red cell distribution width (RDW) in the detection of iron deficiency anaemia in pregnancy within the first 20 weeks of gestation. Bangladesh Med Res Counc Bull. 2011;37:102–5. 10.3329/bmrcb.v37i3.9122 22352230

[15] Tiwari M, Kotwal J, Kotwal A, Mishra P, Dutta V, Chopra S. Correlation of haemoglobin and red cell indices with serum ferritin in Indian women in second and third trimester of pregnancy. Med J Armed Forces India. 2013;69:31–6. 10.1016/j.mjafi.2012.07.016 PMC3862890 24532931

[16] Joerling J, Doll K. Monitoring of iron deficiency in calves by determination of serum ferritin in comparison with serum iron: a preliminary study. Open Vet J. 2019;9:177–84. 10.4314/ovj.v9i2.14 PMC6626149 31360659

